# Neonatal body composition: crossectional study in healthy term singletons in Germany

**DOI:** 10.1186/s12887-019-1837-4

**Published:** 2019-12-12

**Authors:** Cornelia Wiechers, Sara Kirchhof, Lena Balles, Vanessa Avelina, Romy Weber, Christoph Maas, Jan Pauluschke-Fröhlich, Manfred Hallschmid, Hubert Preißl, Andreas Fritsche, Christian F. Poets, Axel R. Franz

**Affiliations:** 1 Department of Neonatology, University Children’s Hospital, Eberhard Karls University, Tuebingen, Calwerstr. 7, 72076 Tuebingen, Germany; 2 Center for Pediatric Clinical Studies, University Children’s Hospital, Eberhard Karls University, Tuebingen, Germany; 3 Department of Obstetrics and Gynecology, University Hospital, Eberhard Karls University, Tuebingen, Germany; 4 Institute for Medical Psychology and Behavioural Neurobiology, Eberhard Karls University, Tuebingen, Germany; 5 German Center for Diabetes Research, Eberhard Karls University, Tuebingen, Germany; 6 Institute for Diabetes Research and Metabolic Diseases of the Helmholtz Center Munich at the Eberhard Karls University, Tuebingen, Germany; 7 Department of Internal Medicine IV, Eberhard Karls University, Tuebingen, Germany

**Keywords:** Infant, Neonatal, Body composition, Air displacement plethysmography, Fat mass

## Abstract

**Background:**

During pregnancy, a variety of factors can influence fetal growth and development. Intrauterine growth may impact on later life and health. Neonatal body composition may be a more sensitive marker for the intrauterine environment than established anthropometric parameters at birth.

**Methods:**

To study neonatal body composition determined by air displacement plethysmography in healthy, term singletons as national reference data, and to establish factors impacting on neonatal body composition in this population. This prospective cross-sectional observational study included 271 healthy, full-term, singletons born between June 2014 and July 2015. Body composition was measured within 96 h of birth using air displacement plethysmography.

**Results:**

Median (Q1, Q2) fat mass / total body mass (BF%) in German singletons was 10.8% (7.7–13.4) and fat free mass (FFM) 2843 g (2606–3099). Female infants had significantly increased BF% compared to male infants (11.2% (8.7–14.0) vs. 9.6% (7.2–12.1)). On multiple regression analysis, BF% and fat mass increased with female gender, maternal pre-pregnancy body mass index, non-smoking mother and parity, whereas FFM increased with male gender and increasing gestational age at birth. Gestational weight gain category, birth mode, and postnatal age at measurement were not associated with BF%, FFM or fat mass.

**Conclusions:**

We generated BF% and FFM centiles for healthy, term, singletons born in Germany; these are similar to those found in other European countries. Infant body composition at birth was associated with modifiable (pre-pregnancy body mass index, smoking), and given factors (gender, gestational age at birth, parity).

## Background

The incidence of obesity in children is rising worldwide. Currently, 17.0% of children in the United States are obese and the prevalence of extreme obesity is 5.8% [1]. In a recent study on German children and adolescents aged 3 to 19 years studied in 2014–2017, the prevalence of overweight was 15.4% and of obesity 5.9%, both increased with age [2]. Obesity in children is relevant for public health, because obese children already have elevated blood pressure and abnormal fasting glucose concentrations [3]. Furthermore, obese children are likely to become obese adults with increased risk of obesity-related complications (e.g., type II diabetes and cardiovascular disease) and mortality [4–6].

Epidemiological studies suggest that inadequate intrauterine supply of nutrients may impact on metabolic health in adulthood [7, 8]. Most studies investigating the relationship between intrauterine growth and later metabolic risk used birth weight alone. It is conceivable, however, that the determination of body composition might be a more sensitive marker for in utero environment and increased neonatal fat mass. It may also be a better indicator of later metabolic risk, as there is considerable variability of neonatal body composition parameters such as fat mass (FM), fat-free mass (FFM) and the proportion of FM divided by total body mass (BF%) in newborns of similar weight and length [9, 10].

There are different methods to determine neonatal body composition (e.g. dual energy x-ray absorptiometry, magnetic resonance imaging or isotope dilution). For about 15 years, air displacement plethysmography (ADP) has been available as a method for rapid, non-invasive, pain-free determination of body composition at relative low cost, providing immediate results without ionizing radiation; thereby making body composition measurements in healthy children acceptable for parents and ethics committees. It has been shown that these measurements are highly reproducible and accurate and therefore suitable even for large epidemiological studies [11, 12]. ADP calculates BF%, FM and FFM according to the two compartment model based on measurements of the infants’ weight and volume.

Reference data for healthy newborns are important as a basis to identify deviations in body composition from the reference standard in special patient groups (e.g., small for gestational age or preterm infants) and to plan intervention studies aiming at improving modifiable pre- and postnatal factors affecting long-term health. For example, the aim of nutritional care of preterm infants is to achieve similar growth as in utero. Due to improvements in nutritional care of preterm infants, weight-gain as in utero is now often achieved [13], but body composition at term equivalent age continues to differ from values found in term-born infants [14]

Differences in body composition between populations of different ethnic and/or socioeconomic background have been reported in adults and children [15, 16] and neonates [12, 17].

We aimed to generate reference data for German Caucasian population for BF%, FM and FFM at birth in healthy, term, singleton infants and to investigate factors influencing body composition.

## Methods

### Participants

This was a prospective, cross-sectional study in a convenience sample of healthy, singleton, term infants (≥37 0/7 weeks gestation) born between June 2014 and July 2015 at Tuebingen University Women’s and Children’s Hospital, Germany. Infants were recruited postnatally by the study team on the puerperal ward if they fulfilled inclusion criteria. Parents were approached preferentially on the day after birth, to enable recuperation from birth. The aim was to address as many parents as possible, restricted by limited availability of the study team. Infants with major congenital anomalies (e.g. congenital heart defects, diaphragmatic hernia, and chromosomal aberrations) or severe diseases (e.g. severe perinatal acidosis, meconium aspiration syndrome) and those born to mothers with pre-gestational or gestational diabetes mellitus were excluded.

Maternal pre-pregnancy body mass index (BMI) (in kg/m^2^) was calculated as pre-pregnancy weight divided by height squared. The following BMI categories were used: underweight (< 18.5); normal weight (18.5–24.9); overweight (25.0–29.9) and obese (> 30) [18].

The Institute of Medicine (IOM) recommendations concerning the recommended gestational weight gain for singleton pregnancies depending on the maternal pre-pregnancy BMI were used to classify weight gain during pregnancy: underweight mothers (recommended gestational weight gain: 12.5–18.0 kg); normal weight mothers (11.5–16.0 kg); overweight mothers (7.0–11.5 kg) and obese mothers (5.0–9.0 kg) [18]. Gestational weight gain below, within or above the recommended range according to maternal pre-pregnancy BMI was classified as “insufficient”, “adequate” and “excessive”, respectively.

### Ethics

The Institutional Review Board approved the study protocol and written informed parental consent was obtained.

### Clinical data collection

Data were collected from maternal health passports and medical records of the mother and her newborn, and parents were asked to fill in a questionnaire. Medical data included age, pre-pregnancy body mass index (BMI), parity, gestational weight gain, smoking during pregnancy and antenatal medical history. Paternal data included age and BMI. Neonatal data included age, sex, birth weight, length and head circumference.

### Anthropometric measures and body composition

The PeaPod Infant body composition system (COSMED, Rome, Italy) is an air displacement plethysmograph and can determine the body composition for infants between 1 and 8 kg body mass. Neonatal anthropometric measures and body composition were determined within 96 h of birth. After weighing, the naked infant was placed in the heated measuring chamber to determine his/her volume. The determination of body volume takes 2 min. BF%, FM, and FFM were calculated by the system as previously described [11, 19]. Body mass was measured to the nearest 0.1 g using the digital scales of the PEAPOD, length to the nearest 0.1 mm using a recumbent, digital infant length board (Ulmer Stadiometer, Busse, Ulm, Germany) and head circumference to the nearest 1 mm using a non-stretchable tape measure.

### Calculation of standard deviation scores (SDS) for weight, length and head circumference

These parameters were computed using LMSgrowth (version 2.14; http://www.healthforallchildren.com/?product=lmsgrowth). The reference population was the British 1990 growth reference (20, 21) fitted by maximum penalized likelihood as described before [20].

### Statistical analyses

Data are presented as mean (standard deviation, SD) if normally distributed, or as median and interquartile range if not. In case that within a table a minority of parameters were normally distributed, the data was nevertheless presented as median (Interquartile range) to improve clarity of presentation. Between group comparisons were performed using a two-sided t-test or ANOVA and post hoc Tukey’s multiple comparison test for normally distributed variables or a Wilcoxon test in non-normally distributed data and a Fisher’s exact test in categorical outcomes. Correlation between normally distributed continuous variables was assessed by linear regression and Pearson correlation coefficient. Associations of potential explanatory variables (gender, parity, maternal smoking, maternal BMI, gestational weight gain category, postnatal age at measurement) with body composition parameters were assessed by multiple linear regression analysis with (manual) backward elimination. Shapiro-Wilk test was used for assessment of normal distribution of data (before ANOVA and t-test) and residuals (for multiple linear regression analyses). Analyses were performed with GraphPad Prism® 8.1.0 (GraphPad Software, San Diego, CA, USA) and the level of significance was *p* < 0.05.

## Results

### Participants

There were 3170 deliveries during the 1-year study period at Tuebingen University Women’s Hospital, Germany; 2649/3170 (83.6%) infants were born with gestational age > 37 weeks, 8/2649 (0.3%) of these died soon after birth or were stillbirths, 80/2649 (3%) infants were twins, 49/2649 (1.9%) infants had severe congenital anomalies and 16/2649 (0.6%) had severe diseases. Of the remaining 2496 singleton, healthy, term infants, 901 (36.1%) families were approached by the study team and 498/901 (55.2%) of these agreed to participate. Thus, 20.0% of eligible infants could be recruited.

In 133 of 498 infants recruited, body composition was not determined due to scheduling difficulties because of early hospital discharge (within 48 h of birth) and unavailability of study personnel on sporadic days. Forty-nine infants were excluded because of a maternal history of pre-gestational or gestational diabetes mellitus, and 45 were excluded for other reasons (e.g., measurement > 96 h after birth (*n* = 20), use of a pacifier or blanket in the test chamber (*n* = 14), or discontinuation of measurement because of agitation or crying (*n* = 3), gestational age at birth < 37 weeks (*n* = 7) or twin pregnancy (n = 1)).

Complete body composition measurements were available for 271 term infants (females *n* = 153).

Demographic data of the study population are shown in Table 1. There was no significant difference in anthropometric parameters between infants born during the study period and not included in the study in comparison with the study population. The admission rate of infants to neonatology was lower in the study group due to recruitment for the study being carried out on the puerperal ward. Furthermore, there was a higher proportion of females in the study and the duration of hospital stay in infants included was slightly longer.

**Table 1:** Characteristics of all singleton, term infants born in Tuebingen during the study period and the study population

		All singleton infants > 37 weeks *n* = 2225*	Study population *n* = 271
Female	n (%)	1099 (49.4%)	153 (56.5%)**
Gestational age at birth (weeks)	Mean (SD)	39.6 (1.2)	39.7 (1.1)
Birth weight (g)	Mean (SD)	3384 (467)	3389 (440)
Birth length (cm)	Mean (SD)	51.0 (2.3)	50.9 (2.3)
Birth head circumference (cm)	Mean (SD)	35.0 (1.6)	34.9 (1.3)
Admission to Neonatology	n (%)	415 (18.7%)	27 (10.0%)**
Duration hospital stay (days)	Median		
All Infants	(Q1, Q3)	3.0 (2.2–3.9)	3.3 (2.4–3.9)
Admission to Neonatology		4.0 (3.2–5.0)	3.7 (3.3–4.6)
Without Admission to Neonatology		2.7 (2.0–3.6)	3.2 (2.4–3.8)**

* All singleton, healthy, term infants > 37 weeks excluding study participants *n* = 2225. Data retrospectively extracted without identifiers from the hospital quality assurance database

** Significantly different from population of infants not-included in the study (*p* < 0.05 by Chi-square and t-test, respectively)

Maternal and paternal characteristics are displayed in Table 2.

**Table 2:** Maternal and paternal demographic data

Demographic Characteristics		Mothers *n* = 271	Fathers *n* = 181
Age at delivery (years)	Mean (SD)	32.5 (5.2)	34.9 (6.4)
Pre-pregnancy weight (kg)	Mean (SD)	65.7 (13.0)	83.7 (12.7)
Height (m)	Mean (SD)	1.67 (0.06)	1.81 (0.07)
Pre-pregnancy BMI (kg/m^2^)	Mean (SD)	23.5 (4.5)	25.1 (4.6)
Gestational weight gain (kg)	Mean (SD)	14.9 (5.2)	
Parity	Mean (SD)	1.6 (0.8)	
Vaginal Delivery	n (%)	175 (64%)	
Pregnancy-induced hypertension	n (%)	7 (3%)	
Familial predisposition for hypertension or diabetes mellitus	n (%)	45 (17%)	
Smoking during pregnancy	n (%)	8 (3%)	
Infertility treatment (ICSI or IVF)	n (%)	14 (5%)	

*Abbreviations*: *BMI* Body mass index, *SD* Standard deviation, *ICSI* Intracytoplasmic sperm injection, *IVF* In vitro fertilization

Median (Q1, Q3) BF% in our population was 10.8% (7.7–13.4) and FFM was 2843 g (2606–3099). For a detailed description of the distribution of the parameters of body composition see Table 3.

**Table 3:** Body composition and characteristics related to body composition measurements

Body Compostion *n* = 271	Min	P 2.5	P 10	P 25	Median	P 75	P 90	P97.5	Max
Fat mass (g)	24	88	161	226	333	443	557	677	894
Fat mass / total body mass (%)	1.0	3.3	5.7	7.7	10.8	13.4	15.8	18.5	21.9
Fat-free mass (g)	1992	2302	2446	2606	2843	3099	3246	3471	4062
Infant Characteristics at BC measurement *n* = 271									
Postnatal age at BC measurement (h)	7.4	11.8	20.0	29.2	42.0	56.0	74.7	82.7	94.1
Weight loss since birth (g)	−23	29	76	132	185	247	298	367	438
Weight at BC measurement (g)	2136	2481	2666	2887	3218	3488	3731	4009	4629

*Abbreviations*: *BC* Body composition

### Univariate analyses

#### Association with gender

Girls had higher BF% and tended to have higher FM than boys, whereas birth weight and FFM was higher in boys (Table 4).

**Table 4:** Summary of Univariate Analyses of Potential Influencing Factors

	n	Birth weight, g Mean (SD)	p*	BF%, % Mean (SD)	p*	FFM, g Mean (SD)	p*	FM, g Mean (SD)	p*
All	271	3389 (440)		10.6 (4.0)		2857 (330)		347 (157)	
Gender			0.0045		0.0012		< 0.0001		0.053
Male	118	3475 (446)		9.7 (3.8)		2957 (328)		326 (149)	
Female	153	3322 (426)		11.3 (4.0)		2780 (311)		363 (161)	
Gestational age (weeks)			< 0.001		0.2276		< 0.0001		0.0116
37.0–37.9	13	2915 (314)		9.5 (3.4)		2471 (162)		266 (121)	
38.0–38.9	51	3140 (407)		10.4 (3.6)		2661 (297)		316 (133)	
39.0–39.9	78	3410 (391)		11.0 (4.5)		2849 (272)		362 (171)	
40.0–40.9	93	3470 (419)		10.1 (3.8)		2949 (327)		336 (148)	
> 41.0	36	3656 (401)		11.6 (3.8)		3054 (291)		412 (167)	
Postnatal age at BC measurement (h)			0.331		0.7539		0.161		0.976
< 24	38	3438 (482)		10.4 (4.2)		2966 (372)		352 (171)	
24.0–47.9	125	3424 (446)		10.4 (4.0)		2883 (316)		346 (159)	
48.0–71.9	76	3345 (415)		10.7 (4.0)		2805 (323)		342 (155)	
72.0–95.9	32	3297 (421)		11.2 (3.7)		2749 (308)		355 (140)	
Pre-pregnangcy BMI (kg/m^2^)			0.0006		0.0089		0.0169		0.0007
< 18.5	7	2939 (413)		8.7 (3.9)		2563 (272)		251 (132)	
18.5–24.9	193	3372 (413)		10.3 (3.8)		2853 (324)		335 (146)	
25.0–29.9	46	3383 (498)		10.7 (4.0)		2842 (361)		351 (155)	
> 30.0	25	3657 (413)		13.0 (4.6)		2996 (271)		459 (197)	
GWG IOM Recommendation			0.013		0.0258		0.0233		0.0102
Insufficient	55	3289 (463)		10.3 (4.2)		2794 (349)		327 (164)	
Adequate	100	3342 (423)		9.9 (3.9)		2832 (314)		319 (145)	
Excessive	116	3477 (432)		11.3 (3.8)		2908 (328)		380 (157)	
Smoking during Pregnancy			0.0014		0.1133		0.0112		0.047
No	255**	3404 (435)		10.7 (4.0)		2866 (328)		351 (157)	
Yes	8	2900 (421)		8.4 (4.1)		2537 (307)		240 (131)	
Parity			0.1111		0.0632		0.2341		0.0413
1	149	3356 (419)		10.1 (3.7)		2845 (324)		325 (145)	
2	82	3473 (425)		11.3 (4.0)		2903 (317)		377 (154)	
≥ 3	40	3339 (529)		11.1 (4.4)		2808 (372)		365 (191)	
Type of delivery			0.4376		0.0563		0.0903		0.2500
Vaginal	175	3404 (423)		10.2 (4.0)		2892 (325)		339 (158)	
Caesarean section	96	3361 (472)		11.2 (3.8)		2793 (331)		361 (153)	

*Abbreviations*: *BC* Body composition, *BMI* Body mass index, *BF%* Proportion of fat mass/total body, *FM* Fat mass, *FFM* Fat free Mass, *GWG* Gestational weight gain, *IOM* Institute of Medicine

* *p*-values by t-test or ANOVA if normally distributed or Wilcoxon-test if not normally distributed

On Tukey multiple comparison post hoc test the following statistically significant differences on multi-group comparison were identified:

Gestational Age (GA, weeks)

Birth weight: GA 37–38 vs. GA 39–40 (*p* = 0.0005), GA 37–38 vs. GA 40–41 (*p* < 0.0001), GA37–38 vs. GA > 41 (*p* < 0.001), GA 38–39 vs. GA 39–40 (*p* = 0.0022), GA 38–39 vs. GA 40–41 (*p* < 0.0001), GA 38–39 vs. GA > 41 (*p* < 0.0001), GA 39–40 vs. GA > 41 (*p* = 0.0221)

FFM: GA 37–38 vs. GA 39–40 (*p* = 0.0002), GA 37–38 vs. 40–41 (*p* < 0.0001), GA 37–38 vs. > 41 (*p* < 0.0001), GA 38–39 vs. GA 39–40 (*p* = 0.0014), GA 38–39 vs.40–41 (*p* < 0.0001), GA 38–39 vs.GA > 41 (*p* < 0.0001)

FM: GA 37–38 vs. GA > 41 (*p* = 0.0309)

Pre-pregnancy BMI category (< 18.5, 18.5–24.9, 25.0–29.9, > 30):

Birth weight: < 18.5 vs. 18.5–24.9 (*p* = 0.0449), < 18.5 vs. > 30 (*p* = 0.0007), 18.5–24.9 vs. > 30 (*p* = 0.0104)

BF%:18.5–24.9 vs. > 30 (*p* = 0.0091)

FFM: < 18.5 vs. > 30 (*p* = 0.011)

FM: < 18.5 vs. > 30 (*p* = 0.0089), 18.5–24.9 vs. > 30 (*p* = 0.0009), 25.0–29.9 (*p* = 0.0246)

Gestational weight gain category (insufficient, adequate, excessive):

Birth weight: insufficient vs. excessive (*p* = 0.0235)

BF%: adequate vs. excessive (*p* = 0.0234)

FFM: insufficient vs. excessive (*p* = 0.0224)

FM: adequate vs. excessive (*p* = 0.0127)

Parity (1,2, ≥3):

FM: 1vs.2 (*p* = 0.0445)

**no smoking status data available in *n* = 8

#### Gestational age at birth

Birth weight (r^2^ = 0.17, *p* < 0.0001), FM (r^2^ = 0.026, *p* = 0.076), and FFM (r^2^ = 0.19, *p* < 0.0001) increased with increasing gestational age on linear regression. For comparisons between subgroups of different gestational ages see also Table 4.

#### Postnatal age

201 of 271 (74.2%) infants were measured between 24 h and 72 h after birth with a median (Q1-Q3) postnatal age of 42 h (29–56). Day of measurement was not associated with changes in BF%, FM or FFM (Table 4).

#### Pre-pregnancy BMI and gestational weight gain

Maternal BMI at the beginning of pregnancy and infants’ BF% showed a minor but statistically significant correlation (r^2^ = 0.05; *p* = 0.0003) and with each maternal BMI point, the offspring’s BF% increased by 0.2%. There was no correlation between absolute maternal weight gain in kg during pregnancy and body composition in linear regression. When the observed gestational weight gain was classified according to the 2009 IOM recommendations taking the pre-pregnancy BMI into account, 20.3% of participants’ mothers gained insufficient weight overall, 36.9% gained adequate weight, and 42.8% gained excessive weight. Excessive weight gain was associated with increased birth weight, BF%, FFM and FM (for between-group comparisons of gestational weight gain classes also see Table 4).

#### Smoking during pregnancy

There were only eight infants (3%) with a history of maternal smoking during pregnancy. Smoking during pregnancy was associated with lower birth weight, lower birth weight SDS (− 0.6 (− 1.5- -0.2) vs. 0.0 (− 0.6–0.6)), lower FM and trends towards lower BF% and FFM (Table 4).

#### Parity and type of delivery

BF% and FM tended to be higher with higher parity (Table 4): first-born infants had a lower BF% than higher-born infants. There was a trend towards higher BF% in infants born by Cesarean section.

#### Multivariate analyses

Multiple linear regression analyses indicated that gender, parity, pre-pregnancy BMI, and smoking were associated with BF% and FM (where FM additionally increased with higher gestational age) whereas FFM was associated with gender and gestational age only (Table 5). In this cohort, measured on day 1 through 4, body composition parameters were not associated with postnatal age.

**Table 5:** Final models of multiple linear regression analyses

Fat Mass [g]				
Influencing Factor	Parameter Estimate	95% Confidence Interval	*p*	r^2^ final model
Intercept	− 956.9	− 1640 - -273.7	0.0062	0.146
Gender[male = 0, female = 1]	51.46	15.55–87.38	0.0051	
Gestational age [weeks]	25.94	8.970–42.91	0.0029	
Parity[n]	34.76	9.461–60.07	0.0073	
Pre-pregnancy BMI[kg/m^2^]	8.189	4.162–12.22	< 0.0001	
Smoking[no = 0, yes = 1]	−99.49	− 185.3 - -13.71	0.0232	
Fat Free Mass [g]				
Intercept	− 2084	− 3438 - -730	0.027	0.260
Gender[male = 0, female = 1]	− 175	− 248.5 - -101.4	< 0.0001	
Gestational age [weeks]	130	96.4 to 164	< 0.0001	
Gestational weight gain category[0 = normal or excessive vs. 1 = insufficient]	− 105	− 195.5 - -14.35	0.0233	
Fat Mass / Total Body Mass (BF%) [%]				
Intercept	4.018	1.423–6.612	0.0025	0.122
Gender[male = 0, female = 1]	1.778	0.8609–2.695	0.0002	
Parity[n]	0.6411	0.0066–1.276	0.0477	
Pre-pregnancy BMI[kg/m^2^]	0.1973	0.0945–0.300	0.0002	
Smoking[no = 0, yes = 1]	−2.344	−4.536 - -0.1512	0.0363	

## Discussion

The aims of this cross-sectional observational study were to establish reference data for body composition by air displacement plethysmography in healthy term singletons for Germany and to investigate various factors potentially influencing body composition.

The infants studied here showed values for median BF% (10.8%) similar to those from other European countries such as Portugal (11.3%) [22], the Netherlands (10.3%) [23], and Ireland (11.1%) [24], but higher values than infants from Australia (BF% 9.2) [12] and Ethiopia (BF% 7.8) [9]. Differences in total body fat between populations of different ethnic background have already been reported in adults and children [15, 16], but little data based on air displacement plethysmography exist in neonates. Furthermore, an Australian study reported that infants of Caucasian mothers showed higher BF% and birthweight in comparison to infants of Asian mothers [12]. While it remains unclear whether the observed differences are truly ethnic (i.e., genetic) or rather economic or nutritional, these factors seem to have an influence on neonatal body composition.

In our study population a significantly higher median BF% (11.2% vs. 9.6%) and lower median FFM (2786 g vs. 2977 g) were found in female infants compared to male infants, and gender was significantly associated with BF%, FM and FFM on multiple regression analysis. Consistent with this, the SCOPE pregnancy study, a large population-based study in Ireland, including 786 term infants confirmed differences in body composition between female and male neonates with girls having a higher BF% and lower FFM [25]. In a cross-sectional Australian study including 599 term infants, gender showed the strongest association with neonatal BF%, followed by maternal ethnicity [12]. Gender is known to be a major determinant of body composition throughout life; girls and adult women also have a higher BF% and lower FFM than their male counterparts [26, 27].

Besides ethnic factors and gender, increasing gestational age is associated with differences in body composition: Hawkes et al. described a significant and linear increase in BF% with increasing gestational age [25], but this was not confirmed in this German population. In this cohort of term infants, we found that BF% showed no significant difference between gestational age groups, and gestational age was only significantly associated with FM and FFM on multivariate analysis.

In our study with measurement of body composition within the first 96 h, postnatal age at measurement was not associated with BF%, FM or FFM. In agreement with our results, there was no association of BF% with postnatal age in the SCOPE study [25], whereas Roggero et al. [28] in a longitudinal study on 28 breastfed, term infants described a higher loss of BF% during the initial weight loss period of the first 5 days after birth compared to the FFM.

BF% and FM were significantly associated with pre-pregnancy maternal BMI. Our results are consistent with previous studies using ADP. In a large pre-birth cohort study, the Healthy Start study (Colorado, USA), Starling et al. showed positive and independent associations of pre-pregnancy BMI with neonatal adiposity measures [29]. Pereira-da-Silva et al. also found that pre-pregnancy overweight was positively associated with offspring weight, BMI and FFM; additionally in male infants also with fat mass [22]. Contrary to this, Eriksson et al. could not find any change in body composition in relation to pre-pregnancy BMI, but weight and BMI of the infants correlated with maternal BMI before pregnancy.

In our study, there was no correlation between absolute maternal gestational weight gain and body composition but a positive association between excessive weight gain by IOM recommendations and neonatal BF%. Additionally, inappropriately low weight gain was associated with decreased FFM in this German cohort. An altered body composition in infants born to mothers with excessive gestational weight gain was also found in the Healthy Start study [29]: gestational weight gain exceeding recommendations was associated with higher neonatal FM and FFM but not BF% compared to adequate weight gain during pregnancy [29].

The association of body composition parameters with pre-pregnancy BMI and gestational weight gain category reflect the impact of intrauterine environment on offspring obesity risk.

There is evidence that obese infants and children are more likely to become obese adults, with an increased risk of characteristic complications (e.g. diabetes and cardiovascular disease) and increased mortality [4–6]. Body composition shortly after birth is not influenced by postnatal factors (i.e., nutritional) and may therefore enables to study the effects of intrauterine environment and targeted interventions (e.g. maternal diet or physical activity before and during pregnancy).

In this study, only 3% (*n* = 8) of mothers reported smoking during pregnancy, nevertheless multivariate analyses showed a significant association with lower BF% and FM, the magnitude of the effect of smoking exceeding that of gender. The apparent lack of impact on FFM may be due to the small numbers. The KiGGS Wave 2 study reported that 10.9% of German mothers smoked during pregnancy and that the proportion of pregnant women who smoke has declined over the last two decades [30]. Furthermore, they found a distinct social gradient in maternal smoking: the higher the social status, the less likely are pregnant women to smoke (1.6% in high social status vs. 27.2% in low social status) [30]. Unfortunately, socio-economic status is difficult to assess reliably and was not documented in this study, however the population of Tuebingen, a town dominated by its university, is generally characterized by a high educational level. It is well known that prenatal smoking increases the risk of intrauterine growth restriction and thus possibly also body composition. The Healthy Start study found a significant effect of exposure to prenatal smoking with a lower FM and FFM at delivery. In that study, 7% of mothers reported smoking during pregnancy [31] and the exposure to prenatal smoking was also associated with a significantly more rapid postnatal growth and increased FFM in the first 5 months of life, possibly explaining the increased risk of metabolic disease in exposed infants.

Strengths of this study are the rather large sample size which was representative for all neonates born during the study period, and the recruitment rate was 55%, which is within the usual range for studies in neonates shortly after birth. Furthermore, inclusion criteria for the study aimed to minimize confounding factors by investigating singleton, healthy, term newborn infants. Using ADP, a reliable, validated and non-invasive method was used for measuring body composition. Based on our experience with participants in neonatal clinical studies at our institution, we assume that a relatively homogeneous population in terms of socioeconomic, ethnic, and cultural background was studied, but it is a weakness of our study that this was not well documented.

In retrospect, additional information on maternal dietary intake, maternal physical activity during pregnancy, as well as placental weight would have been helpful to further identify important factors associated with body composition at birth.

## Conclusions

This cross-sectional study on body composition of healthy term singletons indicates a median (Q1-Q3) BF% of 10.8% (7.7–13.4%) as reference for contemporary neonates for a German Caucasian population. These data are thus similar to those reported for other European countries. Factors associated with body composition in this study were gender, parity, smoking during pregnancy, pre-pregnancy BMI, gestational age at birth and gestational weight gain category, this information will be helpful for the design of future studies.

## Data Availability

De-identified individual data will not be made available, because trial subjects have not been asked to consent.

## Abbreviations

ADP: Air displacement plethysmography
BC: Body composition
BF%: Proportion of fat mass/total body
BMI: Body mass index
FFM: Fat free mass
FM: Fat mass
GWG: Gestational weight gain
N/A: Not available
SD: Standard deviation
SDS: Standard deviation score
